# Treatment of Municipal Wastewater in a Fixed Aerated Bed: Use of Natural Fibrous Materials

**DOI:** 10.1155/2022/4839913

**Published:** 2022-07-19

**Authors:** Murugesan Manikkampatti Palanisamy, Minar Mohamed Lebbai, M. Venkata Ratnam

**Affiliations:** ^1^Department of Food Technology, Excel Engineering College, Namakkal, Tamil Nadu, India; ^2^Department of Chemical Engineering, Erode Sengunthar Engineering College, Erode, Tamil Nadu, India; ^3^Department of Chemical Engineering, Mettu University, Metu Zuria, Ethiopia

## Abstract

The municipal wastewater may be treated using a number of different types of fixed beds that have a larger surface area. Since the fibrous materials have such a large specific surface area, they are frequently considered to be the best option for greater microbiological support and treatment efficacy. In this research, natural fibre materials such as coir fibre and areca husk were investigated for their potential to function as fixed aerated beds for the treatment of municipal wastewater. During the experiment, variations in the chemical oxygen demand (COD), biological oxygen demand (BOD), total dissolved solids (TDS), and total suspended solids (TSS) of the effluent were used to determine how well the aerated fixed bed work in treating the wastewater. The most efficient operating parameters for the successful treatment of wastewater were determined to be a contact period of 72 hrs, a filter medium depth of 5 cm, and a packing density of 10 kg/m^3^. The reductions in BOD, COD, TDS, and TSS for coir fibre are 55%, 58.8%, 57.8%, and 51.89%, respectively, whereas the reductions for areca husk are 38.3%, 37.78%, 31.76%, and 30.56%, respectively. In the course of this experiment, the coir fibre was discovered to be marginally more effective in comparison to the areca husk.

## 1. Introduction

Water is an essential component of the planet and is one of its most vital substances. Water is essential to the survival of all living things, including plants and animals. The availability of clean water for drinking and several other uses is a major concern for people all around the world. Many people believe that the growth of modern industry and the destruction of the natural environment are two sides of the same coin, which highlights the need of having stricter rules for the management of pollutants [1, 2]. The quality of the water is being put in jeopardy by a number of different variables, some of which include recurrent droughts, seasonal and geographical changes in precipitation, excessive use of groundwater, and an absence of uniform distribution of groundwater [2]. The water that is used for drinking must not include any microbes or contaminants. Monitoring the quality of the water is essential in order to maintain a sanitary and risk-free atmosphere [3, 4]. Wastewater is a word that is used to represent liquid waste that is disposed of by a variety of sources, including households, companies, industries, and agriculture, and which frequently contains toxins as a result of the mixing of wastewater from multiple sources [5]. Wastewater is a word that is used to represent liquid waste that is disposed of by a variety of sources, including households, companies, industries, and agriculture.

It is imperative that the wastewater collected from a variety of sources be thoroughly cleaned and disinfected before being released back into the environment. A problematic situation will develop in the event that adequate plans for the collection, treatment, and disposal of all of the garbage produced in the city or town are not put into place [4, 5]. For this reason, it is very necessary that all of the city's wastewater be collected, treated, and disposed of in a risk-free manner in order to protect the health and safety of the residents of the town or city. The inadequate treatment of wastewater before it is released into the environment poses a risk to modern civilization because it creates conditions that are hazardous for both humans and the environment [6]. The elimination of any negative effects, whether they are on human health or the surrounding environment, is the major objective of the treatment of municipal wastewater. The removal of organic waste and nutrients from the effluent is accomplished using a combination of chemical, biological, and physical processes [7].

The conventional treatment systems for this kind of situations may not be feasible due to economic constraints. The treatment of wastewater using anaerobic bacteria has gained popularity in recent years as a result of the cheap costs involved and the minimal damage it does to the surrounding environment [8, 9]. There are a few different approaches to get rid of contaminants, but one that has gained widespread acceptance is filtration. This is due to the numerous advantages that filtration has over other traditional technologies, including high selectivity, low energy consumption, and rapid response kinetics [10]. Because of how simple they are to implement, bio-fixed bed filters have been the subject of a significant amount of research in the field of new waste treatment technologies for both municipal and industrial wastewaters. When used, fibrous packing materials have the effect of significantly lowering the organic strength of municipal wastewater. When there is a significant shift in the flow velocity of the wastewater and the concentration of the water, the mechanism of attached growth seems to be more resilient than the phase of suspended growth [11–13].

The use of aerated filter beds is an unrivalled approach for solid separation. Aerobic attached fixed bed bioreactors have been shown to be an excellent solution for the treatment of residential wastewater. The reactors may be made very compact due to their ability to handle a large amount of biomass, making them an attractive choice for use in tiny wastewater treatment plants. One of the most notable advantages of the linked growth process over other types of bio filters is its uniformity over a wide range of effluent flow rates and concentrations [14, 15]. The properties of the packed bed materials used in the reactors determine the structure of the bio films formed. Hence, the current study employs two different natural materials i.e., coconut fibre and areca husk fibre as fixed beds to treat residential wastewater. A comparison analysis was conducted using four parameters: chemical oxygen demand (COD), biological oxygen demand (BOD), total dissolved solids (TDS), and total suspended solids (TSS). X-ray diffraction (XRD) and scanning electron microscopy (SEM) were also used to characterize the effluent.

## 2. Materials and Methodology

### 2.1. Materials

Coir and Areca husk were gathered in the southern part of Tamil Nadu, India. These are natural fibre materials that are widely available and have a wide range of applications.

### 2.2. Sample Collection

**Figure 1:**
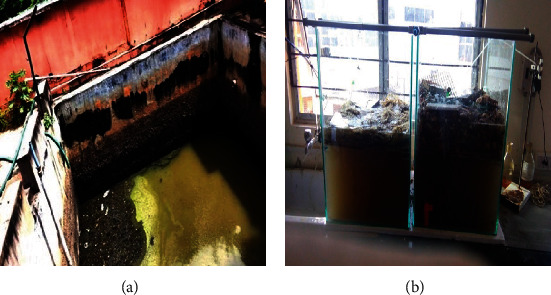
(a) Schematic images of sample collection channel. (b) Fixed bed for effluent treatment.

The discharged wastewater was collected every 24 hrs for 15 days from the drainage channel of Mettur Dam, Salem District, Tamil Nadu, India, as indicated in Figure 1(a) and filtered using Whatman No. 1 filter paper. Furthermore, the filtered material was held under aerobic conditions until microorganism growth was achieved. The microorganisms were immediately employed in the studies once they had grown. The effluent was analyzed using Scanning Electron Microscopy (SEM), and X-ray Diffraction Spectroscopy (XRD).

### 2.3. Experimental Investigations

This method makes use of bio-filters based on trickling bed filters, which treat wastewater by passing it through a filter media containing beds. Inorganic compounds in wastewater decompose owing to biological activity, resulting in a reduction in wastewater characteristics. Two beds made of 6 mm plastic containers with dimensions of 45 cm × 45 cm × 60 cm are used in the present study. The two compartments are set aside for batch operations, in which coir fibre and areca husk are poured to a depth of 5 cm. To eliminate gathered particles, a sludge outlet is provided at the bottom of the compartments. Among the accessories used are mesh, outlets, vent pipes, and taps. Aerators were used in both compartments to maintain steady dissolved oxygen levels. It is seen in Figure 1(b). The compartments are filled with distilled water first, then with the collected wastewater (1 L). The reactors were then aerated for three days until biomass growth was seen in both. The initial and final wastewater parameters, such as pH, COD, BOD, TDS, and TSS, are tested for the samples flowing from the outlet using standard water and wastewater inspection processes.

## 3. Results and Discussions

**Figure 2:**
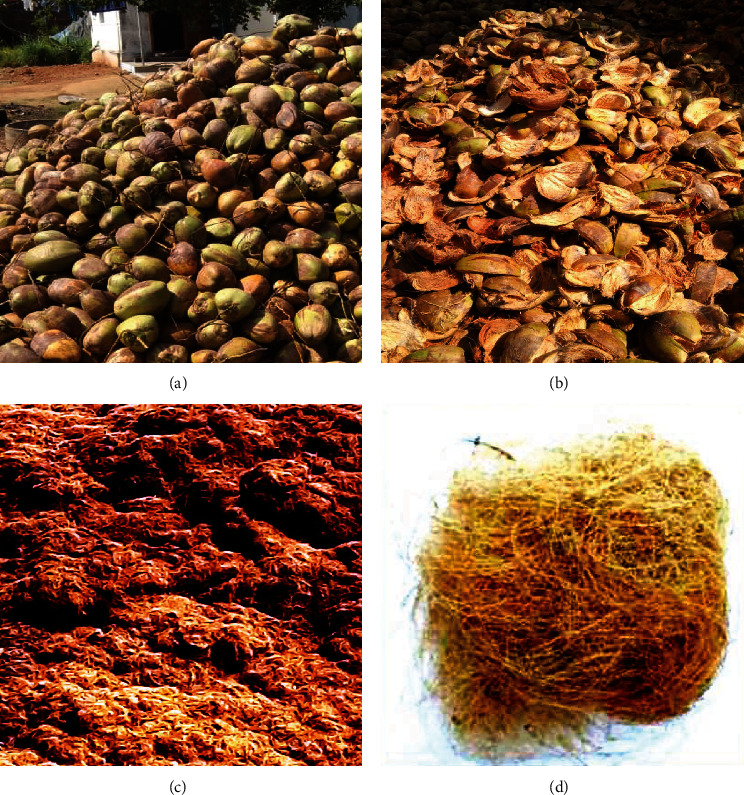
Schematic images of sample collection. (a) Primary husk. (b) Shells for backing support. (c) Areca husk fibre. (d) Coir fibre.

Coir fibre and Areca husk were employed as bio-filters in this experimental study to treat municipal sewage. Natural coir fibre's low density, biodegradability, and low cost make it beneficial for a variety of applications such as roofing sheets, filter media, aggregate agent, reinforced composite material, cement boards, and so on. As a result, this research focuses on the feasibility of employing bio-filters such as coir fibre and areca husk as filter media in fixed aerated beds for the treatment of municipal wastewater using bio aerated bed filters.  Table 1 refers to the general characteristics of both the materials collected [15]. Hence, the coir fibre and areca husk are suitable for use as filter media in a fixed aerated bed (Figures 2(a)-2(d)).

### 3.1. Structural Analysis of the Raw Effluents

**Figure 3:**
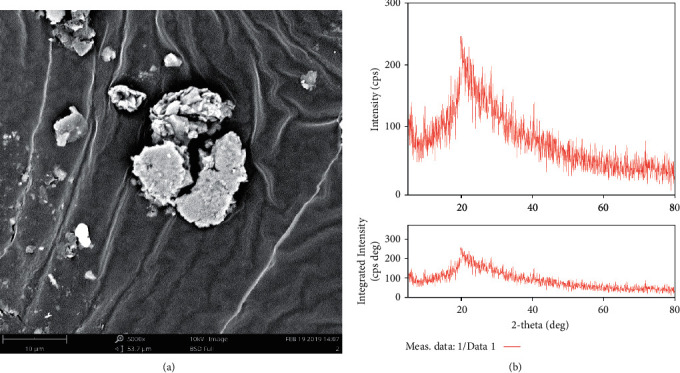
(a) SEM Image of the raw effluent. (b) XRD Spectrum of the raw effluent.

The SEM image of the raw effluent is shown in Figure 3(a), in which the raw effluent with 5 *μ*m magnification seems to have a defined spherical shape with an irregular morphological structure. There is some physical destruction observed in the morphology, which inferred the formation of biomass growth in the effluent. The XRD spectrum of raw effluent was recorded on a Bruker D8 powder. When X-rays with periodic atom configurations contact lattice planes, they scatter selectively in specified directions, producing in high intensity peaks.  Figure 3(b) showed a single peak in the raw effluent with a 2*θ* value of 21.06. The highest intensity at this angle was 384, indicating an amorphous structure. The average particle size was 12 nm, with the apparent decrease in particle size (due to the increased surface area) promoting microbe multiplication.

### 3.2. Removal Efficiency Using Coir as Bio-Filter

The treatment of wastewater by aerated beds using Coir as filter media was investigated and their removal efficiency in BOD, COD, TDS, and TSS was tabulated in Table 2. The coir filter bed was investigated with packing density (5 kg/m^3^ and 10 kg/m^3^) and depth (5 cm and 10 cm).

**Figure 4:**
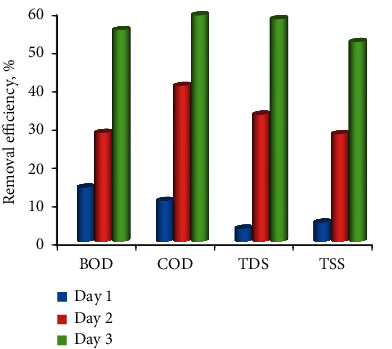
Graphical representation of BOD, COD TDS, and TSS removal using coir fibre with varying the time periods.

BOD, COD, TDS, and TSS in wastewater were calculated at 120 mg/L, 442 mg/L, 976 mg/L, and 422 mg/L, respectively (Table 1, Figure 4). After 24 hrs of interaction, their removal efficiency was 14.1%, 10.6%, 3.38%, and 4.97%. After 48 hrs, the removal efficiency of BOD, COD, TDS, and TSS was found to be 28.3%, 40.53%, 33.3%, and 27.96%, respectively, and after 72 hrs, it was found to be 55.8%, 58.8%, 57.8%, and 51.893%, indicating that the removal efficiency increased with increasing contact period.

#### 3.2.1. Removal Efficiency Using Areca Husk as Bio-Filter

The treatment of wastewater by aerated beds using areca husk as filter media was investigated and their removal efficiency in BOD, COD, TDS, and TSS was tabulated in Table 3. The areca husk filter bed was investigated with packing density (5 kg/m^3^ and 10 kg/m^3^) and depth (5 cm and 10 cm).

**Figure 5:**
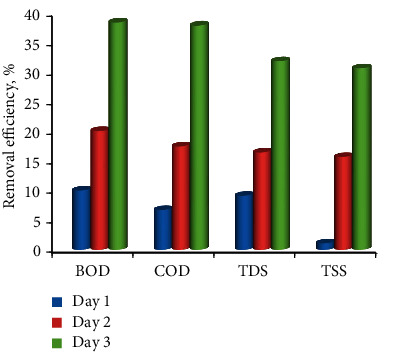
Graphical representation of BOD, COD TDS, and TSS removal using Areca husk with varying the time periods.

Table 3 represents the amount of BOD, COD, TDS, and TSS in raw wastewater were estimated at 120 mg/L, 442 mg/L, 976 mg/L, and 422 mg/L, respectively, also shown in Figure 5. After 24 hrs of contact period, their removal efficiency was found to be 10%, 6.7, 9.11%, and 1.1%, respectively, similarly, after 48 hrs contact period their removal efficiency was 20%, 17.42%, 16.39%, and 15.63%, respectively, and at the end of 72 hrs, it was found to be 38.3%, 37.78%, 31.76%, and 30.56%, respectively. It seems that the removal efficiency increased with increasing their contact period.

### 3.3. Factors Affecting the Treatment of Effluents Using Bio-Filters

#### 3.3.1. Effect of Type of Fibrous Materials

**Figure 6:**
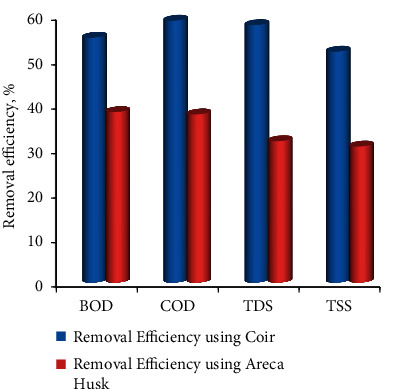
Effect of filter media (fibrous materials) types for the treatment of wastewater.

The wastewater has various contaminants in the form of organic and inorganic material; these have been treated via two natural biological filters, namely, Coir and Areca Husk fibres with 5 cm depth and 10 kg/m^3^ packing density. The removal efficiencies using coir filter media was found as higher removal efficiencies than Areca husk fibres. At the contact period of 72 hrs, the removal efficiencies using Coir were 55%, 58.8%, 57.8%, and 51.89%, and using Areca husk was 38.3%, 37.78%, 31.76%, and 30.56% for the BOD, COD, TDS, and TSS, respectively, its shown in Figure 6.

#### 3.3.2. Effect of Filter Media Depth

**Figure 7:**
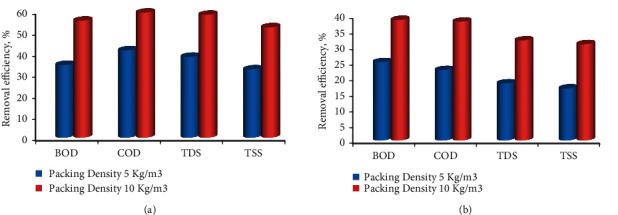
Effect of (a) Coir filter media depth (b) Areca filter media depth for the treatment of wastewater.

In order to optimize the filter media depth, the effluent was fed into fixed aerated beds with two different depths viz. 5 cm and 10 cm. The depth in filter media impacts the removal efficiencies; however, the filter media depth of 5 cm was found as optimum depth with treating 55%, 58.8%, 57.8%, and 51.89% of BOD, COD, TDS, and TSS, respectively, using coir fibres, and furthermore, the removal efficiencies decreased in increasing depth to 10 cm with treating 34.07%, 38.12%, 34.96%, and 29.54% of BOD, COD, TDS, and TSS using coir fibres. Similar trends were observed in using Areca Husk fibres, in the filter media depth of 5 cm the removal efficiencies in BOD, COD, TDS, and TSS were found 38.3%, 37.78%, 31.76%, and 30.56%, respectively, and 10 cm the removal efficiencies were 24.12%, 22.89%, 17.65%, and 14.18%, respectively. It is shown in Figures 7(a)-7(b).

#### 3.3.3. Effect of Packing Density

**Figure 8:**
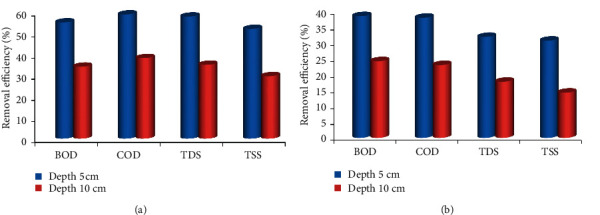
Effect of (a) Coir filter media density and (b) Areca Husk filter media density for the treatment of wastewater.

Aerated beds were used to experiment with the effluent to determine the packing density. Densities of 5 kg/m^3^ and 10 kg/m^3^ were used to test the packing density. For the 10 kg/m^3^ packing density, the removal efficiencies for BOD, COD, TDS, and TSS were 55%, 58.8%, 57.8%, and 51.89%, respectively, while only very minimal reductions were found in the 5 kg/m^3^ packing density, with 34%, 41%, 38%, and 32.14%. For packing density of 10 kg/m^3^, similar observations were made in Areca husk fibres, with removal efficiencies of 38.3%, 37.78%, 31.76%, and 30.56%. For the same packing density, reductions in BOD, COD, TDS, and TSS were measured, with reductions of 24.87%, 22.36%, 18.14%, and 16.54%, respectively. The packing density of 10 kg/m^3^ was shown to be the most effective for removing the greatest amount of contaminants from effluents. It is depicted in Figures 8(a)-8(b). For the purpose of maximizing the contact duration, both compartments were aerated continuously until biomass growth was attained for 3 days in each compartment (72 hrs). The removal efficiency for BOD and COD using coir were 14.1% and 10.6%, respectively, whereas the removal efficiencies for BOD and COD using areca husk were only around 28.3% and 40.5%, respectively, during 24 hrs of contact time. At 72 hrs, the removal efficiency of BOD and COD for coir filter media was 55% and 58.8%, respectively, whereas the removal efficiencies for Areca husk fibres were 38.3% and 37.78%. According to the data, it appears that the removal effectiveness rose as the contact time between the samples was increased.

### 3.4. SEM Analysis Samples for Coir Filter Media and Areca Husk Filter Media after Treatment of Wastewater

**Figure 9:**
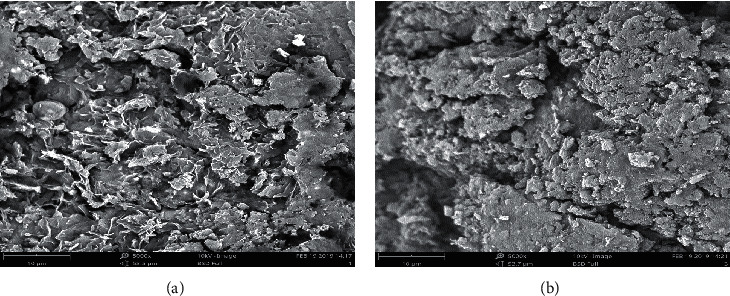
SEM images of the (a) Coir filter media and (b) Areca Husk bio materials after the treatment.

The attached biomass on the material surface was captured by the SEM analysis done for the Coir filter media and Areca Husk bio materials after the treatment (Figures 9(a)-9(b)). The bio films were found to be densely containing microorganisms.

## 4. Conclusions

The treatment of municipal wastewater via natural fibrous materials as fixed aerated beds was demonstrated experimentally. From this investigation, significant reductions in BOD, COD TDS, and TSS were obtained using both the Coir and Areca husk fibres. However the maximum removal efficiencies were 55%, 58.8%, 57.8%, and 51.89% found in Coir fibrous material and comparatively lower reductions were achieved in using Areca husk filter media i.e., 38.3%, 37.78%, 31.76%, and 30.56%. The contact period of 72 hrs, filter media depth of 5 cm and packing density of 10 kg/m^3^ was demonstrated as optimum operating conditions for the maximum removal of BOD, COD TDS, and TSS. The foul in the filter due to the fibrous decomposition causes filtration rate and thus makes operation malfunctions. The spent fibres in filter media may after be used as organic manure because it contains nutrient in rich amount. Since the fibrous materials are economically viable and their treatment efficiencies are relatively high, this system may be recommended for municipal and industrial wastewater treatments. The treated wastewaters are then used for gardening, and other domestic activities such as floor washes and vessel cleaning.

## Figures and Tables

**Table 1:** Characteristics of Coir fibre and Areca husk.

	Coir fibre	Areca husk
Cellulose	46%	50–60%
Lignin	44.6%	7–8%
Moisture	4–5%	4–5%
Hemi cellulose	0.25%	20–30%
Ash	3%	1–3%

**Table 2:** The removal efficiency of wastewater using Coir fibre as filter media.

Parameters	Raw effluent (mg/L)	Coir fibre as filter media at optimum conditions					
		Day 1 (mg/L)>	Day 2 (mg/L)	Day 3 (mg/L)	Removal efficiency (%)		
					Day 1	Day 2	Day 3
BOD	120	103	86	54	14.1	28.3	55
COD	442	395	263	182	10.6	40.5	58.8
TDS	976	943	654	411	3.38	33	57.8
TSS	422	401	304	213	4.97	27.96	51.89

**Table 3:** The removal efficiency of wastewater using Areca husk as filter media.

Parameters	Raw effluent (mg/L)	Areca husk as filter media at optimum conditions					
		Day 1 (mg/L)	Day 2 (mg/L)	Day 3 (mg/L)	Removal efficiency (%)		
					Day 1	Day 2	Day 3
BOD	120	108	96	74	10	20	38.3
COD	442	412	365	275	6.7	17.42	37.78
TDS	976	887	816	666	9.11	16.39	31.76
TSS	422	417	356	293	1.1	15.63	30.56

## Data Availability

The data used to support the study are included in the paper.

## Nomenclature

BOD: Biological Oxygen Demand
COD: Chemical Oxygen Demand
FTIR: Fourier Transform Infra-Red Spectroscopy
GAC: Granular Activated Carbon
IL: Ionic Liquid
SEM: Scanning Electron Microscopy
TDS: Total Dissolved Solids
TSS: Total Suspended Solids
XRD: X-ray Diffraction Spectroscopy
